# Carbohydrate Counting as a Low-Cost Strategy Associated With Better Glycemic Control in Children With Type 1 Diabetes Mellitus in Resource-Constrained North India: A Cross-Sectional Mediation Analysis

**DOI:** 10.7759/cureus.114355

**Published:** 2026-08-11

**Authors:** Anuradha Sharma, Aashish Sethi, Nishant Sharma, Utkarsh Sharma, Shantanu Shubham

**Affiliations:** 1 Pediatrics, Great Ormond Street Hospital for Children NHS Foundation Trust, London, GBR; 2 Pediatrics, Graphic Era Deemed to be University, Dehradun, IND; 3 Pediatrics, Uttar Pradesh University of Medical Sciences, Saifai, IND; 4 Pediatrics, Shri Guru Ram Rai Institute of Medical & Health Sciences, Dehradun, IND

**Keywords:** carbohydrate counting, glycemic control, hba1c, pediatric diabetes, qolid, quality of life, resource-limited setting, type 1 diabetes mellitus

## Abstract

Background: Children with type 1 diabetes mellitus (T1DM) in India face a dual burden of disease-related stress and socioeconomic constraint, with insulin pumps and continuous glucose monitors (CGMs) unaffordable for most families. Carbohydrate (carb) counting is a low-cost, education-based self-management skill linked to improved glycemic control in other populations. We hypothesized that carb counting is associated with better quality of life (QoL) in children with T1DM primarily through improved glycemic control, and tested this pathway using a cross-sectional mediation analysis at a tertiary center in North India.

Methodology: In this hospital-based, cross-sectional study, 50 children with T1DM (age 2-18 years) attending the pediatric endocrinology outpatient clinic of a tertiary care hospital in Dehradun, Uttarakhand, were enrolled. QoL was assessed using the Quality-of-Life Instrument for Indian Diabetes patients (QOLID; 34 items across eight domains). Glycated hemoglobin (HbA1c), self-monitored blood glucose, insulin regimen, and carb-counting practice were recorded. Associations were analyzed using independent-samples t-tests, Pearson correlation, and simple linear regression, with sensitivity analyses (Welch's t-test, Mann-Whitney U), exploratory multivariable linear regression, and Baron-Kenny mediation analysis (Sobel test and bootstrap confidence intervals) used to test whether HbA1c mediates the carb-counting-QoL association. p<0.05 was considered significant.

Results: The mean QOLID score was 82.08±9.06%; 19 (38%) children had impaired QoL (score <80%). Financial worries were the lowest-scoring domain (69.50±17.00), impaired in 44 (88%) children, despite 47 (94%) being on basal-bolus insulin therapy and only one (2%) child using an insulin pump. HbA1c showed the strongest negative correlation with total QoL (r=-0.675, p<0.001). Thirty (60%) children practiced carb counting; they had significantly lower HbA1c (7.27±1.02% vs 8.96±2.50%, p=0.0017) and higher total QOLID scores (148.37±12.46 vs 139.40±18.43, p=0.045) than those who did not. Mediation analysis showed that HbA1c fully mediated the carb counting-QoL association: the indirect effect via HbA1c was significant (a×b=9.26, Sobel z=2.87, p=0.0041; bootstrap 95% CI: 2.74-16.82), while the direct effect of carb counting on QoL was negligible and non-significant after accounting for HbA1c (c'=-0.29, p=0.938), with an estimated 103% of the total effect statistically mediated through glycemic control.

Conclusions: Carb counting was associated with better QoL primarily, and statistically fully, through its association with better glycemic control rather than through an independent pathway -- a mechanistically coherent, testable explanation for how a free, education-based skill could plausibly improve QoL in settings where insulin pumps and CGMs remain unaffordable. Financial worry remained the most impaired QoL domain despite near-universal use of low-cost insulin regimens, underscoring the broader economic burden of care. As this mediation pattern is statistical rather than temporally confirmed, prospective or interventional studies are needed before structured carb-counting education can be recommended as an evidence-based strategy in resource-constrained pediatric diabetes care.

## Introduction

India carries one of the largest pediatric burdens of type 1 diabetes mellitus (T1DM) worldwide, with an estimated 15,900 new cases diagnosed annually and reported incidence rates ranging from 3.7 to 31.9 per 100,000 population [1,2]. While national attention to diabetes largely centers on adult type 2 disease, children with T1DM confront a uniquely demanding disease course: lifelong insulin dependence, rigid meal timing, repeated glucose testing, fear of needles, and the psychosocial burden of a chronic illness during formative years [3,4]. These challenges are magnified in India by low disease awareness, limited parental health literacy, and, critically, financial constraint, which shapes nearly all management decisions families make [3].

Quality of life (QoL), a multidimensional construct spanning physical, psychological, and social well-being, is now recognized as a core outcome in diabetes care rather than merely a byproduct of glycemic control [5,6]. Children and adolescents with T1DM consistently report poorer health-related QoL than their healthy peers, and poor QoL has, in turn, been linked to worse treatment adherence and glycemic outcomes [7-9]. Yet QoL research in Indian pediatric T1DM remains sparse, and most available instruments were developed and validated in Western populations with markedly different healthcare and family structures. The Quality-of-Life Instrument for Indian Diabetes patients (QOLID) was developed specifically to address this gap, incorporating a dedicated “financial worries” domain that captures a dimension of disease burden rarely measured elsewhere [10].

This financial dimension is not incidental. Diabetes technology -- insulin pumps, continuous glucose monitors (CGMs), and hybrid closed-loop systems -- has transformed T1DM care globally, but in India these devices remain financially out of reach for the overwhelming majority of families; government schemes do not routinely subsidize insulin, pumps, or sensors for children with T1DM [11,12]. Even the direct costs of “minimal” conventional care -- insulin, syringes, glucometer strips, and laboratory monitoring -- can consume over half of household income in low-resource Indian families, forcing many to borrow money or forgo other necessities [12,13]. In this context, families and clinicians need low-cost, scalable strategies that may improve outcomes without adding to this burden.

Carbohydrate (carb) counting is one such potentially cost-effective strategy. Rather than requiring costly hardware, it is a dietary self-management skill -- taught by a physician, nurse, or dietitian -- through which patients match prandial insulin doses to the carb content of meals using an individualized insulin-to-carb ratio [14], a recommendation retained in the updated 2022 ISPAD consensus guidelines, which further emphasize adapting carb-counting education to regional diets, foods, and available resources [15]. Indian implementation experience with this approach is also accumulating: a randomized trial of basic carb counting in children and young adults at a North Indian tertiary center found the method feasible and more acceptable to families than routine nutrition education, although overall glycemic benefit at 12 weeks was confined to subgroups, underscoring that the intensity and quality of implementation may determine effectiveness [16]. Multiple international studies and meta-analyses have linked carb counting to improved glycemic control and QoL in children and adults with T1DM [17-19], and emerging Indian data support similar benefits, including reduced diabetes-related distress [20,21].

Building on our institutional QoL dataset in children with T1DM, this cross-sectional study tests the specific hypothesis that carb counting is associated with better QoL primarily through improved glycemic control, using mediation analysis alongside conventional univariable and multivariable methods. Clarifying this pathway has practical relevance: if carb counting's benefit for QoL operates chiefly through glycemic control, structured carb-counting education becomes a mechanistically coherent, low-cost candidate strategy for children with T1DM growing up in financially constrained households, where insulin pumps and other advanced technologies remain, for now, largely inaccessible.

## Materials and methods

Study design and setting

This hospital-based, cross-sectional, descriptive study was conducted in the pediatric endocrinology outpatient clinic of a tertiary care teaching hospital in Dehradun, Uttarakhand, North India. Patients were enrolled consecutively over an eight-month period after obtaining clearance from the institutional ethics committee.

Participants

Children and adolescents aged 2-18 years with a confirmed clinical and biochemical diagnosis of T1DM, on treatment for at least six months, were eligible; children diagnosed less than six months prior were excluded to allow stabilization of the insulin regimen. To ensure reliable ascertainment of carb-counting status specifically, eligibility additionally required that a child's treatment approach -- either practicing or not practicing carb counting -- had remained stable for this same six-month period, with at least monthly outpatient follow-up throughout, cross-checked against the institutional clinic data entry register; children without this consistent follow-up history were not included. Written informed consent was obtained from parents/guardians, and the study was approved by the institutional ethics committee of the study site. All eligible children attending the clinic during the eight-month enrollment window were approached consecutively; 50 children fulfilling eligibility criteria consented and were enrolled. Given the low reported prevalence of pediatric T1DM in the region (approximately 18 per 100,000 population) [1], a formal precision-based sample size calculation was not feasible within the study period, and this cohort should be regarded as a consecutive sample rather than one derived from an a priori power calculation. A post hoc assessment of detectable effect sizes, given this sample size, is presented in the Limitations.

Data collection

Demographic data (age, sex, religion, region, and socioeconomic status by modified Kuppuswamy scale), anthropometric measurements (weight, height, body mass index (BMI), and corresponding Z-scores per Indian Academy of Pediatrics 2015 growth charts), disease characteristics (age at onset, duration, presenting symptoms, and associated complications), and treatment details (insulin regimen, daily insulin dose, number of boluses, and carb-counting status) were recorded on a structured proforma.

Carb counting was taught to families by the treating pediatric endocrinologist and a dedicated type 1 diabetes educator at the study institute, delivered across multiple structured sessions using standardized educational modules. Carb-counting status (use of a quantified insulin-to-carb ratio for prandial dosing, as distinct from fixed-dose or correction-only dosing) was recorded at enrollment as part of the structured proforma. As above, this status was required to have been stable for at least six months and was corroborated by a minimum of monthly outpatient follow-up during this period, cross-checked against the institutional clinic data entry register, rather than being based on a single unverified self-report. Formal, quantitative competency testing of carb-counting accuracy (e.g., calculation-accuracy quizzes) was not performed as part of this study, so ascertainment still relied on clinical documentation of practice rather than directly observed or tested skill; this residual limitation is discussed further in the Limitations.

Glycemic control was assessed using glycated hemoglobin (HbA1c), measured by high-performance liquid chromatography (BIO-RAD D-10) after a minimum of six months on the same treatment regimen. Seven-day self-monitored blood glucose records (diabetes diary) were used to calculate average fasting and average whole-day blood glucose.

QoL assessment

QoL was measured using the QOLID, originally developed and validated in adult Indian patients with diabetes by Nagpal et al. [22] and subsequently modified for the pediatric age group by Kumar et al. [10]. The original validation study describes the QOLID as the first instrument specifically developed, translated, and psychometrically validated for health-related and diabetes-specific QoL assessment in Indian patients with diabetes [22], in contrast to prior Indian pediatric diabetes QoL studies, which relied on internationally developed generic or diabetes-specific instruments (e.g., PedsQL, DAWN Youth QoL, and WHO-5) validated in Western populations rather than an Indian cultural, linguistic, and socioeconomic context. The QOLID incorporates domains relevant to the Indian setting, including a dedicated financial worries domain, and underwent forward-backward translation and psychometric validation (Cronbach's alpha 0.894) in adults [22] before its pediatric adaptation [10] was adopted here. It consists of 34 items across eight domains (three generic health-related domains and five disease-specific domains, including financial worries), each scored on a five-point Likert scale (1-5, higher=better QoL, maximum raw score 170); domain scores were standardized to a 0-100 scale, and the overall QOLID score was the mean of the eight standardized domain scores.

The cutoff used to classify “impaired” versus “good” QoL (80%, corresponding to a raw score of >140/170) was not re-derived from the present cohort; it was adopted from Kumar et al.’s independent pediatric validation study of 97 Indian children with T1DM [10], in which this threshold, together with its associated sensitivity (78%) and specificity (65%), was established using receiver operating characteristic (ROC) curve analysis within that separate cohort. Because we applied this previously published, externally derived cutoff to our sample rather than deriving and evaluating a new cutoff within our own n=50 dataset, the specific same-sample circularity risk raised in peer review does not apply to our use of it. We note that the original source study’s own ROC analysis appears to have used a median-based internal classification within its cohort, a characteristic of that source study’s methodology rather than of the present analysis. As this externally sourced cutoff has not been independently re-validated by us in the current sample, our primary analyses nonetheless rely on the continuous QOLID score, and the good/impaired categorization is reported only descriptively; no conclusion in this manuscript depends on this categorical cutoff. Because higher scores denote better QoL, a negative correlation between a clinical variable and the continuous QOLID score indicates that higher values of that variable are associated with worse QoL, and vice versa.

Children older than 10 years completed the questionnaire directly; parents served as proxy respondents for younger children. This mixed self-report/parent-proxy approach, necessitated by the wide age range studied, may introduce measurement heterogeneity, since parent-proxy and child self-report of QoL can diverge (see Discussion); this could not be formally adjusted for in our analysis. Parent-proxy and child self-report scores were not analyzed separately by respondent type, as the resulting subgroups would have been too small for an adequately powered comparison. The QOLID was administered bilingually (English and Hindi), the Hindi version generated by forward translation with independent back-translation to confirm equivalence.

Statistical analysis

Continuous variables are expressed as mean±SD, and categorical variables as frequencies and percentages. Independent-samples t-tests and chi-square/Fisher's exact tests compared QoL between groups, including by carb-counting status. Associations between total and domain-level QOLID scores and demographic, clinical, and glycemic variables were explored using Pearson's correlation coefficient (r) and simple linear regression, which are mathematically equivalent for a single continuous predictor; correlation coefficients (r) and their associated p-values are reported throughout. Most reported associations are univariable. Two exploratory multivariable linear regression models (predicting total QOLID score and HbA1c, respectively, each with 4-6 covariates given the modest sample size of n=50) and a formal mediation analysis of the carb counting-QoL-HbA1c pathway were additionally performed and are reported separately in Results; findings from these adjusted models should still be regarded as exploratory given the sample size relative to the number of covariates.

Normality of key continuous variables was assessed using the Shapiro-Wilk test, and homogeneity of variance between carb-counting groups was assessed using Levene's test; where assumptions were violated, Welch's t-test and the Mann-Whitney U test were used as sensitivity analyses alongside the primary Student's t-test. Mediation of the carb counting-QoL association by HbA1c was tested using the Baron-Kenny approach (total effect c, path a: carb counting→HbA1c, path b: HbA1c→QoL controlling for carb counting, direct effect c’: carb counting→QoL controlling for HbA1c), with the indirect effect (a×b) tested for significance using the Sobel test and a 5,000-replicate percentile bootstrap 95% confidence interval. No correction for multiple testing (e.g., Bonferroni) was applied given the exploratory nature of this study; findings should be interpreted as hypothesis-generating rather than confirmatory. A p-value <0.05 was considered statistically significant. All primary analyses were performed using SPSS version 20.0 (IBM Corp., Armonk, NY); mediation and sensitivity analyses were performed in Python (SciPy; Python Software Foundation, Fredericksburg, VA, US).

## Results

Of 50 enrolled children (31, 62% boys; 19, 38% girls), the mean age at enrollment was 9.66±4.09 years, and the mean age at diagnosis was 7.01±3.94 years, with a mean disease duration of 2.66±1.99 years. Most participants were from urban households (40, 80%) and from lower-middle to upper socioeconomic strata (28, 56% in the “upper” group). Twenty-five (50%) children had presented in diabetic ketoacidosis (DKA) at diagnosis; the remaining 25 (50%) presented with classic symptoms of polyuria, polydipsia, and weight loss without DKA. The large majority (47, 94%) were on a basal-bolus insulin regimen; 2 (4%) children used premixed insulin, and only 1 (2%) child used an insulin pump. Mean total daily insulin requirement was 0.99±0.32 units/kg/day. Comorbid autoimmune conditions were present in 5 (10%) children: 3 (6%) with celiac disease and 2 (4%) with autoimmune hypothyroidism; 1 additional child had a pre-existing diagnosis of autism. Diabetes-related complications were present in 8 (16%) children: diabetic nephropathy in 3 (6%) and ophthalmologic complications (refractive error in 3, cataract in 2) in 5 (10%). Thirty (60%) children practiced carb counting in combination with correction-dose adjustment, while 35 (70%) used correction doses alone for at least part of their bolus calculation (Table 1).

**Table 1:** Baseline demographic, anthropometric, and treatment characteristics of the study population (n=50) Values are presented as mean±SD for continuous variables and n (%) for categorical variables. Socioeconomic status was classified using the modified Kuppuswamy scale, and BMI Z-scores were derived from the Indian Academy of Pediatrics 2015 growth charts. BMI, body mass index; DKA, diabetic ketoacidosis; HbA1c, glycated hemoglobin; SD, standard deviation.

Characteristic	Value
Age at enrollment, years (mean±SD)	9.66±4.09
Age at diagnosis, years (mean±SD)	7.01±3.94
Duration of diabetes, years (mean±SD)	2.66±1.99
Male sex, n (%)	31 (62.0)
Urban residence, n (%)	40 (80.0)
Upper socioeconomic status (Kuppuswamy), n (%)	28 (56.0)
Normal BMI Z-score (≥-2 SD), n (%)	47 (94.0)
Presentation in DKA, n (%)	25 (50.0)
Basal-bolus insulin regimen, n (%)	47 (94.0)
Insulin pump use, n (%)	1 (2.0)
Daily insulin requirement, U/kg/day (mean±SD)	0.99±0.32
HbA1c, % (mean±SD)	7.95±1.93
Practicing carbohydrate counting, n (%)	30 (60.0)
Local site complications (lipodystrophy/infection), n (%)	12 (24.0)

QoL

The mean overall QOLID score was 82.08±9.06% (on a continuous scale). Using the externally sourced 80% cutoff described in Methods, 31 (62%) children were classified as having good QoL and 19 (38%) impaired QoL; this categorical classification is reported for descriptive purposes only and was not used to derive the correlational findings below. Physical endurance was the best-scoring domain (94.93±8.91), while financial worries was, by a wide margin, the worst-scoring domain (69.50±17.00); 44 (88%) children scored below 80% on this domain alone, the highest rate of impairment of any domain, exceeding even diet satisfaction (41, 82% impaired) and treatment satisfaction (31, 62% impaired) (Table 2).

**Table 2:** QOLID domain scores and proportion of children with impaired quality of life (QoL<80%) in each domain (n=50) QoL, quality of life; QOLID, Quality-of-life Instrument for Indian Diabetes patients; SD, standard deviation.

QOLID domain	Mean±SD (0-100 scale)	Impaired QoL, n (%)
Physical endurance	94.93±8.91	4 (8.0)
Symptom botherness	91.20±14.03	13 (26.0)
Role limitation – physical health	85.93±11.90	16 (32.0)
General health	84.67±11.91	19 (38.0)
Emotional/mental health	83.20±12.93	23 (46.0)
Treatment satisfaction	75.10±13.61	31 (62.0)
Diet satisfaction	72.10±12.74	41 (82.0)
Financial worries	69.50±17.00	44 (88.0)
Overall QOLID score	82.08±9.06	19 (38.0)

Correlates of QoL

Total QOLID score correlated strongly and negatively with HbA1c (r=-0.675, p<0.001) and with average pre-breakfast (r=-0.366, p=0.009) and average whole-day blood glucose (r=-0.408, p=0.003); these glycemic measures showed the strongest associations of any variables studied. Children with good QoL (by the exploratory cutoff) had a lower mean HbA1c than those with impaired QoL (7.05±1.21% vs 9.5±1.95%, p<0.00001).

At the domain level, HbA1c correlated significantly and negatively with every QOLID domain except financial worries, which was the sole domain showing no significant correlation with HbA1c (r=-0.133, p=0.356) (Table 3).

**Table 3:** Correlation of HbA1c with total and domain-level QOLID scores in children with type 1 diabetes mellitus (n=50) Correlation coefficients (r) were calculated using Pearson's product-moment correlation, with two-tailed tests of significance; a p-value <0.05 was considered statistically significant. HbA1c, glycated hemoglobin; QOLID, Quality-of-life Instrument for Indian Diabetes patients.

QOLID domain	Correlation coefficient (r)	p-value
Role limitation – physical health	-0.548	<0.001
Physical endurance	-0.619	<0.001
General health	-0.578	<0.001
Treatment satisfaction	-0.562	<0.001
Symptom botherness	-0.531	<0.001
Financial worries	-0.133	0.356
Emotional/mental health	-0.540	<0.001
Diet satisfaction	-0.410	0.003
Total QOLID score	-0.675	<0.001

Age (r=-0.063, p=0.664), age at diagnosis (r=-0.050, p=0.732), and disease duration (r=-0.046, p=0.752) showed no significant correlation with total QoL. However, several expected positive correlations emerged among the demographic and glycemic variables themselves, supporting the internal consistency of the dataset: age correlated positively with age at diagnosis (r=+0.873, p<0.001) and disease duration (r=+0.310, p=0.029); HbA1c correlated positively with average pre-breakfast glucose (r=+0.613, p<0.001) and average whole-day glucose (r=+0.688, p<0.001), which in turn correlated strongly with each other (r=+0.786, p<0.001); and disease duration correlated positively with average whole-day glucose (r=+0.281, p=0.048). The eight QOLID domains were also consistently and positively inter-correlated with one another and with the total score (r=+0.30 to +0.86, most p<0.01); the financial worries domain again stood apart, correlating only weakly with role limitation -- physical health, general health, and emotional health (r=+0.28 to +0.30) and not significantly with physical endurance, treatment satisfaction, symptom botherness, or diet satisfaction.

Local site complications (lipodystrophy/injection-site infection) were associated with poorer QoL (mean total score: 134.25 vs 148.11, p=0.006), as was lower socioeconomic status (138.82 vs 149.46, p=0.015). Sex, religion, region, BMI Z-score, DKA at presentation, and number of daily insulin boluses showed no significant association with QoL.

Carb counting: univariable comparisons

Children practicing carb counting had significantly higher total QOLID scores than those who did not (148.37±12.46 vs 139.40±18.43, p=0.045, Student's t-test) (Table 4). The effect was most pronounced in the physical endurance domain (29.23±1.46 vs 27.35±3.60, p=0.013), with favorable but non-significant trends across most other domains.

Note: Tables 2, 3 report standardized QOLID domain scores (0-100 scale); Table 4 reports raw, unstandardized domain scores as originally analyzed by carb-counting status, with maxima differing by domain according to item count. Raw and standardized scores should not be compared directly.

**Table 4:** Comparison of raw QOLID domain scores between children practicing and not practicing carbohydrate counting (n=50) Values are mean±SD of raw (unstandardized) domain scores. Groups were compared using the independent-samples t-test (two-tailed, df=48); a p-value <0.05 was considered statistically significant. Carb, carbohydrate; QOLID, Quality-of-life Instrument for Indian Diabetes patients.

QOLID domain	Carb counting: Yes (n=30)	Carb counting: No (n=20)	t-value	p-value
Role limitation – physical health	26.40±3.50	24.85±3.56	1.524	0.134
Physical endurance	29.23±1.46	27.35±3.60	2.576	0.013
General health	13.07±1.44	12.15±2.13	1.818	0.075
Treatment satisfaction	15.40±2.37	14.45±3.15	1.215	0.230
Symptom botherness	13.87±2.15	13.40±2.06	0.765	0.448
Financial worries	14.10±3.56	13.60±3.22	0.506	0.616
Emotional health	21.40±2.84	19.90±3.64	1.635	0.109
Diet satisfaction	14.90±2.35	13.70±2.72	1.660	0.103
Total raw score	148.37±12.46	139.40±18.43	2.056	0.045

HbA1c was significantly lower in children practicing carb counting than in those who did not (7.27±1.02% vs 8.96±2.50%, p=0.0017); diabetes duration was significantly shorter (2.02±1.70 vs 3.67±2.04 years, p=0.0033); and daily insulin boluses were significantly more frequent (3.41±0.68 vs 2.84±0.83, p=0.0125) in carb counters. Total daily insulin dose (p=0.79) and age (p=0.19) did not differ between groups (Table 5).

**Table 5:** Clinical and demographic comparison by carbohydrate-counting status in children with type 1 diabetes mellitus (n=50) Values are mean±SD. Groups were compared using the independent-samples t-test (two-tailed, df=48); t-values and p-values are shown, and a p-value <0.05 was considered statistically significant. HbA1c, diabetes duration, and daily insulin boluses differed significantly between groups, whereas total daily insulin dose and age did not; the shorter diabetes duration and more frequent boluses among children practicing carbohydrate counting are relevant to the confounding-by-indication discussion in the text. Carb, carbohydrate; HbA1c, glycated hemoglobin; SD, standard deviation.

Variable	Carb counting: Yes (n=30)	Carb counting: No (n=20)	t-value	p-value
HbA1c, %	7.27±1.02	8.96±2.50	-3.323	0.0017
Diabetes duration, years	2.02±1.70	3.67±2.04	-3.088	0.0033
Daily insulin boluses, n	3.41±0.68	2.84±0.83	2.598	0.0125
Total insulin dose, U/kg/day	1.01±0.24	0.98±0.43	0.264	0.7931
Age, years	9.04±4.12	10.60±3.98	-1.329	0.1901

Sensitivity analyses and assumption checks

Total QOLID score, HbA1c, and diabetes duration were non-normally distributed (Shapiro-Wilk p≤0.006), and Levene's test showed unequal variance between carb-counting groups for total QOLID score (p=0.025) and HbA1c (p<0.001). Repeating the comparisons with methods robust to these violations, the HbA1c difference remained significant (Welch's t p=0.0087; Mann-Whitney U p=0.011), but the total QOLID score difference did not (Welch's t p=0.076; Mann-Whitney U p=0.148).

Multivariable analysis

In an exploratory multivariable model predicting total QOLID score from carb-counting status, HbA1c, socioeconomic status, age, diabetes duration, and BMI Z-score (n=50, adjusted R²=0.496, F(6,43)=9.04, p<0.001), only HbA1c and socioeconomic status were independently associated with QoL; carb counting was not (Table 6).

**Table 6:** Exploratory multivariable linear regression predicting total QOLID score in children with type 1 diabetes mellitus (n=50) The model included carbohydrate-counting status (yes/no), HbA1c, socioeconomic status (modified Kuppuswamy scale, ordinal), age, diabetes duration, and BMI Z-score as predictors of the total QOLID raw score (adjusted R²=0.496, F (6,43)=9.04, p<0.001). Unstandardized regression coefficients (β), standard errors, t-values, and two-tailed p-values are shown; a p-value <0.05 was considered statistically significant. Given the modest sample size relative to the number of covariates, this model should be regarded as exploratory. BMI, body mass index; carb, carbohydrate; HbA1c, glycated hemoglobin; QOLID, Quality-of-life Instrument for Indian Diabetes patients; SE, standard error.

Predictor	β (unstandardized)	SE	t-value	p-value
Carbohydrate counting (yes/no)	0.43	2.28	0.189	0.851
HbA1c, %	-2.85	0.56	-5.065	<0.001
Socioeconomic status (ordinal 1-5)	2.64	1.02	2.585	0.013
Age, years	0.22	0.24	0.917	0.364
Diabetes duration, years	0.12	0.56	0.205	0.838
BMI Z-score	1.22	0.75	1.618	0.113

In a second exploratory model predicting HbA1c from carb-counting status, diabetes duration, age, and socioeconomic status (n=50, R²=0.273), carb counting remained independently associated with lower HbA1c after adjustment; duration, age, and socioeconomic status were not independently significant (Table 7).

**Table 7:** Exploratory multivariable linear regression predicting HbA1c from carbohydrate-counting status and covariates in children with type 1 diabetes mellitus (n=50) The model included carbohydrate-counting status (yes/no), diabetes duration, age, and socioeconomic status (modified Kuppuswamy scale, ordinal) as predictors of HbA1c (%) (R²=0.273). Unstandardized regression coefficients (β), standard errors, t-values, and two-tailed p-values are shown; a p-value <0.05 was considered statistically significant. Given the modest sample size relative to the number of covariates, this model should be regarded as exploratory. Carb, carbohydrate; HbA1c, glycated hemoglobin; SE, standard error.

Predictor	β (unstandardized)	SE	t-value	p-value
Carbohydrate counting (yes/no)	-1.17	0.57	-2.048	0.046
Diabetes duration, years	0.15	0.15	1.041	0.304
Age, years	0.06	0.06	1.010	0.318
Socioeconomic status (ordinal 1-5)	-0.48	0.26	-1.843	0.072

Mediation analysis: does HbA1c explain the carb counting-QoL association?

Given that carb counting was robustly associated with lower HbA1c (path a), and HbA1c was strongly associated with QoL (above), we formally tested whether HbA1c mediates the association between carb counting and total QOLID score using the Baron-Kenny approach (Table 8). The total effect of carb counting on QoL (path c) was significant (β=8.97, p=0.045, matching the raw-score comparison in Table 4). Carb counting was significantly associated with lower HbA1c (path a: β=-1.69, p=0.0017), and HbA1c remained strongly associated with QoL when controlling for carb counting (path b: β=-5.49, p<0.001). Once HbA1c was included, the direct effect of carb counting on QoL (path c’) was reduced to near zero and lost all statistical significance (β=-0.29, p=0.938). The indirect effect through HbA1c (a×b=9.26) was significant by the Sobel test (z=2.87, p=0.0041) and by a 5,000-replicate bootstrap 95% confidence interval that excluded zero (2.74 to 16.82), and accounted for an estimated 103% of the total effect -- consistent with full statistical mediation (Table 8).

**Table 8:** Mediation analysis of HbA1c as a statistical mediator of the association between carbohydrate counting and total QOLID score (Baron-Kenny approach, n=50) Path c, the total effect of carbohydrate counting on QoL; path a, the association of carbohydrate counting with HbA1c; path b, the association of HbA1c with QoL controlling for carbohydrate counting; and path c′, the direct effect of carbohydrate counting on QoL controlling for HbA1c. The indirect effect (a×b) was tested using the Sobel test and a 5,000-replicate percentile bootstrap 95% confidence interval; the proportion of the total effect mediated was estimated as (a×b)/c (values may exceed 100% when the direct effect is opposite in sign to the indirect effect). Unstandardized coefficients are shown; a p-value <0.05 was considered statistically significant. Because all variables were measured concurrently in this cross-sectional study, this mediation is statistical and does not establish temporal precedence or causation. Carb, carbohydrate; CI, confidence interval; HbA1c, glycated hemoglobin; QoL, quality of life; QOLID, Quality-of-life Instrument for Indian Diabetes patients.

Path	Description	β/effect	p-value
c	Total effect: carb counting → QoL	8.97	0.045
a	Carb counting → HbA1c	-1.69	0.0017
b	HbA1c → QoL (controlling for carb counting)	-5.49	<0.001
c’	Direct effect: carb counting → QoL (controlling for HbA1c)	-0.29	0.938
a×b	Indirect effect via HbA1c (Sobel test)	9.26	0.0041
—	Bootstrap 95% CI for indirect effect (5,000 reps)	2.74 to 16.82	—
—	Proportion of total effect mediated	≈103%	—

## Discussion

This cross-sectional study in children with T1DM from a tertiary center in North India provides evidence for a specific, mechanistically coherent pathway: carb counting was associated with better QoL primarily, and statistically fully, through its association with better glycemic control, rather than through an association with QoL that is independent of HbA1c. This central finding, together with our observation that financial worry was the single most impaired QoL domain despite near-universal use of low-cost insulin regimens, is discussed below.

Our overall QOLID score (82.08±9.06) closely mirrors the mean of 84.1±6.88 reported in the original Indian validation cohort [10], and the strength of the HbA1c-QoL correlation (r=-0.675) is consistent with several international and Indian cohorts showing that poorer metabolic control correlates strongly with poorer QoL in pediatric T1DM [8,23,24].

Our mediation analysis (Table 8) provides a coherent explanation for a pattern that would otherwise appear contradictory: carb counting showed a robust univariable and multivariable-adjusted association with lower HbA1c (persisting after adjustment for duration, age, and socioeconomic status, β=-1.17%, p=0.046), and a nominally significant but fragile univariable association with total QOLID score (p=0.045 by Student’s t-test, but not significant by Welch’s t-test or Mann-Whitney U). Rather than treating these as two disconnected, partly contradictory findings, formal Baron-Kenny mediation analysis shows they fit together: the entire total effect of carb counting on QoL (c=8.97) is statistically accounted for by its indirect path through HbA1c (a×b=9.26, Sobel p=0.0041, bootstrap 95% CI: 2.74-16.82), while the direct path (c’=-0.29, p=0.938) is negligible. In other words, carb counting does not appear to improve QoL through some independent psychological or behavioral mechanism (e.g., a sense of mastery or control) once its glycemic benefit is accounted for; essentially all of its statistical association with QoL is consistent with operating through improved glycemic control. This is, we believe, a more mechanistically satisfying and clinically actionable finding than an unexplained direct association would have been, because it identifies a concrete, measurable intermediate target (HbA1c) through which an intervention could plausibly act.

This mediation pattern should still be interpreted cautiously. Our data are cross-sectional: HbA1c and QoL were measured at the same time point as carb-counting status, so temporal precedence (that carb counting preceded improved HbA1c, which in turn preceded better QoL) cannot be established, and reverse pathways (e.g., families coping better psychologically finding it easier to sustain carb counting and thereby achieving better control) remain possible. Families who practice carb counting also had shorter disease duration and more daily insulin boluses, consistent with broader engagement in intensive self-management, of which carb counting may be one marker among several rather than the sole active ingredient; this residual confounding cannot be fully excluded even after our multivariable adjustment. Formal, prospective mediation analysis, ideally within an interventional trial, is needed to confirm temporal ordering.

A second, distinct observation concerns the domain-level pattern in QoL scores. Financial worries scored lowest of all eight domains and was impaired in 44 (88%) children, even though 47 (94%) of our cohort was managed on standard, comparatively low-cost basal-bolus multiple daily injection (MDI) therapy rather than expensive pump technology; only 1 (2%) patient used an insulin pump. Financial distress in our cohort therefore cannot be explained by spending on advanced technology, since almost none of our patients had access to it; it likely reflects the baseline cost of “minimal” T1DM care itself, consistent with financial worries being the only QOLID domain not significantly correlated with HbA1c -- that is, financial worry appears to sit largely outside the glycemic-control pathway described above and represents a structurally distinct unmet need.

A large North Indian survey similarly found families spent a median of over INR 55,000 annually on glucose monitoring, insulin, and laboratory testing alone, with 30% spending over half their household income on diabetes care [13]; global Life for a Child data likewise show minimal annual supply costs can consume 12%-370% of per-capita gross national income in less-resourced countries, with meters/strips, not insulin, the largest driver [12]. Government schemes in most Indian states, including ours, do not routinely subsidize insulin or consumables for pediatric T1DM. Insulin pump therapy, though associated with better glycemic control and QoL in several studies, remains inaccessible for most Indian children; small NGO-industry pilot programs have extended pump access to some underprivileged children with encouraging results [25,26] but are not yet available at a national scale.

Our finding that carb counting was robustly associated with lower HbA1c, and that this association statistically accounts for its relationship with QoL, is consistent with international interventional evidence: a systematic review and meta-analysis of nine pediatric studies found that carb counting improved glycemic control across most included studies [17], and a more recent meta-analysis quantified this as an average HbA1c reduction of 0.94% versus fixed-dose approaches [18]. A narrative review similarly reported improved glucose control across pediatric studies using mobile carb-counting applications [19]. An Indian randomized controlled trial from New Delhi found that children practicing carb counting had significantly lower diabetes-related distress than those on a fixed meal plan [20,21], and a second Indian randomized trial reported a substantial HbA1c reduction (10.6±3.0% to 8.4±1.5% over six months, p<0.00001) with a structured carb-counting protocol [27]. Our cross-sectional mediation analysis adds a QoL-relevant mechanistic layer to this interventional literature: it suggests that where carb counting improves QoL, it likely does so via the same glycemic pathway these trials targeted directly, rather than via an independent effect on QoL.

QoL was self-reported by children older than 10 years but obtained via parent proxy for younger children; parent-proxy and child self-report of QoL can diverge in the broader literature [24], and this mixed-respondent approach, necessitated by the wide age range studied, may have introduced measurement heterogeneity not adjusted for in our analysis or mediation model. Parent-proxy and child self-report scores were not analyzed separately by respondent type: the resulting subgroups would have been small and unevenly distributed across carb-counting groups, leaving any such comparison underpowered, so respondent type remains a potential unmeasured source of heterogeneity in both the univariable and mediation analyses.

Socioeconomic status was independently associated with QoL in multivariable analysis (p=0.013), consistent with broader evidence that socioeconomic status shapes QoL across pediatric chronic diseases generally, not only diabetes [28]. Local site complications (lipodystrophy, injection-site infection) -- a largely preventable consequence of inadequate injection-site rotation, itself often linked to reuse of needles or syringes to conserve cost -- were associated with poorer QoL in univariable analysis, suggesting that low-cost interventions such as structured injection-technique education may warrant further study in this population.

Taken together, our findings support a specific, testable model for this setting: carb counting, financial worry, and QoL are linked chiefly via glycemic control, with financial distress operating as a separate, structural burden largely outside this pathway. This positions structured carb-counting education as a mechanistically coherent, low-cost candidate strategy for improving glycemic control, and thereby QoL, in children with T1DM where insulin pumps and CGM remain inaccessible -- a more specific and actionable hypothesis than a generic “carb counting improves QoL” claim would have been. Given the cross-sectional design and modest sample size, we present this mediation model as a hypothesis for prospective and interventional confirmation, with HbA1c as a pre-specified mediator, rather than as a basis for immediate policy recommendations.

Limitations

This study has several limitations. The sample was a consecutive series of 50 children attending a single center over an eight-month period rather than one derived from a formal a priori precision-based calculation. A post hoc assessment indicates approximately 80% power to detect a between-group Cohen's d of 0.81 or larger (n=30 vs n=20, α=0.05 two-tailed); the observed effect for total QOLID score by carb-counting status (d≈0.59) corresponds to only about 54% power, indicating the study was underpowered even for this significant finding, consistent with its non-robustness to sensitivity analysis described above. For the full cohort (n=50), 80% power was available only for correlations of |r|≥0.39 or larger; non-significant correlations below this threshold (e.g., age, age at diagnosis, and disease duration with QoL, all |r|<0.07) may reflect true near-null associations, but smaller true correlations could plausibly have been missed.

Although carb-counting education was delivered by the treating pediatric endocrinologist and a diabetes educator using standardized modules across multiple sessions, and status was required to be stable for at least six months and corroborated by monthly follow-up cross-checked against institutional records (reducing, though not eliminating, recall or misclassification bias relative to an unverified single-time-point self-report), formal quantitative competency testing of carb-counting accuracy was not performed, so ascertainment still relied on clinical documentation of practice rather than directly observed or tested skill. Hypoglycemia frequency, including severe and nocturnal episodes, was not systematically captured, in part owing to the cost of continuous glucose monitoring in this population. Because hypoglycemia is among the strongest drivers of diabetes-related distress and QoL in children with T1DM, and because carb counting could plausibly influence hypoglycemia risk itself, the absence of these data is a particularly important limitation when interpreting the carb counting-QoL association and the mediation model reported here: a hypoglycemia-related pathway can be neither quantified nor excluded. Our two multivariable models are exploratory, given a sample size (n=50) that is modest relative to the number of covariates (4-6), and the results require replication in larger cohorts; residual and unmeasured confounding, including confounding by indication for carb counting, cannot be fully excluded even after adjustment. The cross-sectional design precludes establishing temporal precedence for the mediation pathway reported (carb counting → HbA1c → QoL); HbA1c and QoL were measured concurrently, so reverse or bidirectional pathways cannot be excluded, and our mediation analysis is statistical rather than a confirmed causal chain. No correction for multiple comparisons was applied across the many univariable tests reported. The 80% QoL cutoff was adopted from an external source study rather than re-derived or re-validated within the present sample, and its performance characteristics (sensitivity, specificity) reflect those of the source cohort rather than ours; primary analyses therefore rely on continuous QOLID scores rather than this categorical cutoff. Because only one child used an insulin pump, a direct comparison between pump and MDI regimens was not possible. Finally, this single-center Indian cohort may limit generalizability to other regions and healthcare systems.

## Conclusions

In this cross-sectional cohort of Indian children with T1DM, carb counting was associated with QoL primarily, and statistically fully, through its association with better glycemic control: mediation analysis showed a significant indirect effect via HbA1c (Sobel p=0.0041) accounting for essentially all of the total effect, while the direct effect of carb counting on QoL was negligible and non-significant. Socioeconomic status was independently associated with QoL, and financial worry, rather than physical or technological limitation, was the most impaired QoL domain, despite near-universal reliance on standard, lower-cost basal-bolus insulin regimens. Given the cross-sectional design and modest sample size, this mediation pattern is statistical rather than a confirmed causal chain, and should be regarded as hypothesis-generating. Prospective or interventional studies, with pre-specified, temporally-ordered mediation analysis, are needed to confirm whether structured carb-counting education improves glycemic control and, through it, QoL for children with T1DM growing up in financially constrained households, before it can be recommended as an evidence-based component of routine pediatric diabetes care. Pending such confirmation, the key clinical and policy implication of these findings remains that structured, low-cost educational strategies such as carb counting, deliverable within routine outpatient visits without recurring hardware costs, represent the most immediately scalable candidates for evaluation and implementation in settings where advanced diabetes technology is unaffordable, while broader financial support mechanisms for families are pursued.
